# Bistable Morphing Composites for Energy-Harvesting Applications

**DOI:** 10.3390/polym14091893

**Published:** 2022-05-05

**Authors:** Ammar Elsheikh

**Affiliations:** Production Engineering and Mechanical Design Department, Faculty of Engineering, Tanta University, Tanta 31527, Egypt; ammar_elsheikh@f-eng.tanta.edu.eg

**Keywords:** bistable morphing composites, actuating techniques, energy harvesting, mathematical modeling, artificial intelligence

## Abstract

Bistable morphing composites have shown promising applications in energy harvesting due to their capabilities to change their shape and maintain two different states without any external loading. In this review article, the application of these composites in energy harvesting is discussed. Actuating techniques used to change the shape of a composite structure from one state to another is discussed. Mathematical modeling of the dynamic behavior of these composite structures is explained. Finally, the applications of artificial-intelligence techniques to optimize the design of bistable structures and to predict their response under different actuating schemes are discussed.

## 1. Introduction

Polymer-based composites have been widely used in many industrial and engineering applications instead of other materials due to their enhanced properties [1,2]. The main advantages of polymeric composites are availability, ease of processing, high strength, excellent chemical resistance, and low cost [3,4]. They have good flexural strength, tensile strength, impact strength, Young’s modulus, compressive strength, and high dimensional stability [5]. They have been reported as excellent alternatives to metals and ceramics in automotive, aerospace, building or medical applications [6,7,8]. 

Bistable morphing composites (BMCs) have received significant attention in recent decades as new innovative composite structures due to their outstanding mechanical properties, high space utilization, low weight, and their fabulous behavior in energy-harvesting applications [9,10,11]. They have been widely used in critical engineering applications such as airfoils, automobile structures, de-icing/anti-icing systems, soft robotics, deployable structures, piezoelectric actuation and energy harvesting [12,13,14].

**Figure 1:**
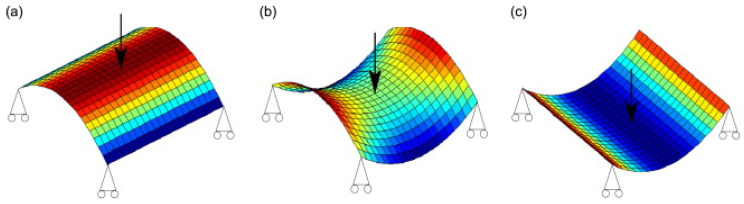
Shapes of bistable sheet: (**a**) first stable saddle shape, (**b**) unstable shape of the sheet, (**c**) second stable saddle shape [24].

The composite structures, such as glass or carbon fibers reinforced polymers (GFRP and CFRP), exhibit two main natural equilibrium states without applying any external force to preserve their shape at one equilibrium state [15]. Thus, a bistable composite sheet can settle at one state without any external loading. These structures undergo large deformations between the two equilibrium states with a slight amount of energy [16]. This strange behavior was observed by many researchers, but Hyer [17,18] was the first one to declare that this behavior could be exploited in useful applications. He determined the shape of unsymmetric four-layer laminates cooled down from curing temperature to room-temperature. The stable saddle shapes of the laminates are formed due to the thermal expansion difference between the laminates, which induces thermal residual stresses. As shown in Figure 1, BMCs undergo two stable states (Figure 1a,c) and one unstable state (Figure 1b).

BMC have received considerable attention in various engineering applications due to their excellent stiffness/strength-to-weight, outstanding fatigue characteristics, excellent durability, enhanced damage performance, and design flexibility [19]. To obtain optimal designs of BMC structures, advanced computational tools should be developed. Many theoretical studies are reported in the literature to determine the behavior of BMCs regarding 1D, 2D or 3D models. Analytical and numerical computational approaches were employed to model BMCs [20,21,22]. Finite element simulations and artificial-intelligence tools have been also reported in literature, to overcome the difficulties in solving the nonlinear mathematical models of BMC [23].

In this paper, the application of bistable morphing composites in energy harvesting is reviewed. First, the mathematical modeling of BMCs is introduced. Then, the actuation techniques used to derive BMC structures is discussed. After that, energy harvesting using BMC is presented. Finally, the application of artificial-intelligence tools, such as artificial-neural networks and metaheuristic optimizers, to predict and optimize the behavior of BMC structures is discussed.

## 2. Mathematical Modeling

Many attempts have been reported in literature to model BMC [25,26,27]. Dano and Hyer [25] presented a methodology to obtain the out-of-plane displacement of BMCs after they cooled down from cure temperature to room temperature. The Rayleigh–Ritz method was employed to obtain mathematical expression of the total potential energy using approximated values of the strain fields. Curvatures of BMCs as a function of temperature were determined. In another study, the same authors simplified their model and compared the obtained results from the model with those obtained from force-controlled experiment [26]. Mattioni et al. [28] modified the model by considering more boundary conditions and compared its results with those obtained from finite element analysis. They also carried out a parametric analysis to explore the effects of the laminate thickness and fiber orientation on the BMC behavior. Diaconu et al. [29] analyzed the dynamic behavior of BMCs using Hamilton’s principle and the Rayleigh–Ritz method. They succeeded in obtaining the stable-state displacements of a BMC and predicting the dynamic and static transitions between stable states. The dynamic response of BMCs was defined using two distinct phases: the large-amplitude phase and small-amplitude phase. The mathematical modeling of BMC behavior based on the aforementioned studies will be summarized in this section. The entire virtual work $\;\left(\delta W_{E}\right)$ is given in terms of strain energy $\left(SE\right)$, kinetic energy $\left(KE\right)$ and nonconservative forces work $\left(W_{C}\right)$ using Hamilton’s principle, as follows:

(1) $$\delta W_{E}=\delta \;\overset{t_{1}}{\underset{t_{0}}{\int }}\left(KE+W_{C}-SE\right)dt=0$$

The displacement $\left(k,m,n\right)$ of a point with three-dimensional coordinates $\left(x,y,z\right)$ from the midplane of the sheet is given based on Kirchhoff’s theory as a function of time and midplane displacements $\left(k^{0},m^{0},n^{0}\right)$, as follows:

(2) $$k\left(x,y,z,t\right)=k^{0}\left(x,y,t\right)-z\frac{\delta n^{0}\left(x,y,t\right)}{\delta x}$$

(3) $$m\left(x,y,z,t\right)=m^{0}\left(x,y,t\right)-z\frac{\delta n^{0}\left(x,y,t\right)}{\delta x}\;$$

(4) $$n\left(x,y,z,t\right)=n^{0}\left(x,y,t\right)$$

Strain-displacement relations according to von Kármán formulas are given as functions of the normal strains $\epsilon^{0}={\left[\varepsilon_{x}^{0},\varepsilon_{y}^{0},\gamma_{xy}^{0}\right]}^{T}$ of the midplane and its corresponding curvatures $\Gamma^{0}={\left[\Gamma_{x}^{0},\Gamma_{y}^{0},\Gamma_{xy}^{0}\right]}^{T},\;$ as follows:

(5) $$\varepsilon_{x}=\varepsilon_{x}^{0}+\;z\Gamma_{x}^{0}$$

(6) $$\varepsilon_{y}=\varepsilon_{y}^{0}+\;z\Gamma_{y}^{0}$$

(7) $$\gamma_{xy}=\gamma_{xy}^{0}+\;z\Gamma_{xy}^{0}$$

where

(8) $$\varepsilon_{x}^{0}=\frac{\partial k^{0}}{\partial x}+\frac{1}{2}{\left(\frac{\partial n^{0}}{\partial x}\right)}^{2}$$

(9) $$\varepsilon_{y}^{0}=\frac{\partial m^{0}}{\partial y}+\frac{1}{2}{\left(\frac{\partial n^{0}}{\partial y}\right)}^{2}$$

(10) $$\gamma_{xy}^{0}=\frac{\partial k^{0}}{\partial y}+\frac{\partial m^{0}}{\partial x}+\frac{\partial n^{0}}{\partial x}\frac{\partial n^{0}}{\partial y}\;$$

(11) $$\Gamma_{x}^{0}=-\frac{\partial^{2}n^{0}}{\partial x^{2}}\;$$

(12) $$\Gamma_{y}^{0}=-\frac{\partial^{2}n^{0}}{\partial y^{2}}\;$$

(13) $$\Gamma_{xy}^{0}=-2\frac{\partial^{2}n^{0}}{\partial x\partial y}\;$$

The kinetic energy and strain energy are computed according to the following formulas:

(14) $$\mathrm{KE}=\frac{1}{2}\;\int_{-\frac{a_{y}}{2}}^{\frac{a_{y}}{2}}\int_{-\frac{a_{x}}{2}}^{\frac{a_{x}}{2}}\left\{J_{0}\left({\dot{k}}^{2}+{\dot{m}}^{2}+{\dot{n}}^{2}\right)-2J_{1}\left(\dot{k}\frac{\partial^{2}n^{0}}{\partial x\partial t}+\dot{m}\frac{\partial^{2}n^{0}}{\partial y\partial t}\right)\;+J_{2}\left({\left(\frac{\partial^{2}n^{0}}{\partial x\partial t}\right)}^{2}+{\left(\frac{\partial^{2}n^{0}}{\partial y\partial t}\right)}^{2}\right)\right\}dxdy$$

(15) $$\mathrm{SE}=\frac{1}{2}\;\int_{-\frac{a_{y}}{2}}^{\frac{a_{y}}{2}}\int_{-\frac{a_{x}}{2}}^{\frac{a_{x}}{2}}{\left[\begin{array}{c} F\\ M \end{array}\right]}^{T}{\left[\begin{array}{c} \epsilon^{0}\\ \Gamma^{0} \end{array}\right]}^{T}dxdy$$

where $\;J_{0},J_{1}\;$ and $J_{2}$ are distributed mass factors and they are computed as follows:

(16) $$J_{0}=\int_{-\frac{a_{z}}{2}}^{\frac{a_{z}}{2}}\rho dz;\;\;J_{1}=\int_{-\frac{a_{z}}{2}}^{\frac{a_{z}}{2}}\rho zdz;\;J_{2}=\int_{-\frac{a_{z}}{2}}^{\frac{a_{z}}{2}}\rho z^{2}dz$$

where ρ is the density of the BMC, $F={\left[F_{x},F_{y},F_{xy}\right]}^{T}$ is the force vector and $M={\left[M_{x},M_{y},M_{xy}\right]}^{T}$ is the moment vector.

(17) $$\left\{\left[\begin{array}{c} F_{x}\\ F_{y}\\ F_{xy} \end{array}\right],\left[\begin{array}{c} M_{x}\\ M_{y}\\ M_{xy} \end{array}\right]\right\}=\int_{-\frac{a_{z}}{2}}^{\frac{a_{z}}{2}}\left[\begin{array}{ccc} p_{11} & p_{12} & p_{16}\\ p_{11} & p_{22} & p_{26}\\ p_{16} & p_{26} & p_{66} \end{array}\right]\left(\left[\begin{array}{c} \varepsilon_{x}\\ \varepsilon_{y}\\ \varepsilon_{xy} \end{array}\right]-\left[\begin{array}{c} \mu_{x}\\ \mu_{y}\\ \mu_{xy} \end{array}\right]\Delta KE\right)\left(dz,\;zdz\right)$$

where $p_{ij}$ denotes the stiffness components and $\mu_{x}$, $\mu_{y}$ and $\mu_{xy}$ are the thermal expansion components. 

The work performed on the BMC depends on the nature of external forces as well as their point of action. The displacements of the laminate mid-plane $\left(k^{0},m^{0},n^{0}\right)$ can be expressed in the form of the following nonlinear differential equations by substituting the computed strain energy, kinetic energy, and the computed work performed according to Hamilton’s principle. There is no analytical solution for the obtained displacement equations. Therefore, the Rayleigh–Ritz approach was employed to obtain an approximate analytical solution [25]. Approximated expressions of strain components could be obtained using polynomial expansions, as follows:

(18) $$\varepsilon_{x}^{0}\left(x,y\right)=d_{1}+d_{2}x^{2}+d_{3}xy+d_{4}y^{2}$$

(19) $$\varepsilon_{y}^{0}\left(x,y\right)=d_{5}+d_{6}x^{2}+d_{7}xy+d_{8}y^{2}$$

where $d_{1},d_{2},\ldots ,\;d_{8}$ are polynomial constants.

The out-of-plane displacement is given by:

(20) $$n^{0}\left(x,y\right)=\frac{1}{2}\left(d_{9}x^{2}+d_{10}y^{2}+d_{11}xy\right)$$

where $d_{9}=-\Gamma_{x}^{0}$ and $d_{10}=-\Gamma_{y}^{0}$ denote the curvatures in the plane defined by $\left(x,y\right)$ and $d_{11}=-\Gamma_{xy}^{0}$ denotes the twisting curvature. 

If the curvature variation of boundary conditions is considered, Equation (20) should be modified as follows [26]:

(21) $$n^{0}\left(x,y\right)=d_{00}+d_{10}x+d_{01}y+d_{20}x^{2}+d_{02}y^{2}+d_{11}xy+d_{12}xy^{2}\;+d_{21}x^{2}y+\;d_{22}x^{2}y^{2}$$

(22) $$k^{0}\left(x,y\right)=d_{1}x+\frac{1}{3}d_{2}x^{3}+d_{3}xy^{2}+\frac{1}{2}d_{4}x^{2}y-\frac{1}{6}d_{9}^{2}x^{3}-\frac{1}{4}\;d_{9}d_{11}x^{2}y\;-\frac{1}{8}d_{11}^{2}xy^{2}+d_{12}y+\frac{1}{3}d_{13}y^{3}$$

(23) $$m^{0}\left(x,y\right)=d_{5}y+d_{6}x^{2}y+\frac{1}{3}d_{7}y^{3}+\frac{1}{2}d_{8}xy^{2}-\frac{1}{6}d_{10}^{2}y^{3}-\frac{1}{4}\;d_{10}d_{11}xy^{2}\;-\frac{1}{8}d_{11}^{2}x^{2}y+d_{15}x+\frac{1}{3}d_{14}x^{3}$$

The in-plane shear strain is given by substituting the displacement expression into Equation (10):

(24) $$\begin{array}{ll} \gamma_{xy}^{0}=2\;d_{12}+ & \left(d_{9}d_{10}-\frac{1}{4}d_{11}^{2}+2d_{3}+2d_{6}\right)xy\\ & +\left(\frac{d_{9}d_{11}+2d_{4}}{4}+d_{14}\right)x^{2}+\left(\frac{d_{10}d_{11}+2d_{8}}{4}+d_{13}\right)y^{2} \end{array}$$

In the aforementioned expressions, there are 14 constants that should be computed via the back-substitution technique. The curvatures and strains of the midplane are substituted into the total strain and strain energy expressions.

This mathematical modeling helps to understand and figure out the effects of different aspects on the bistability characteristics of these composites. Nevertheless, there are several uncertainties in the main properties of bistable composites materials that come from the fabrication processes used [30]. These uncertainties affect not only the analytical results but also the experimental ones. They may also impair structural reliability and the corresponding dynamic responses [31]. Thus, to augment the performance and preserve the reliability of composite laminates, these uncertainties should be carefully investigated. Brampton et al. [27] investigated the effect of geometrical uncertainties, environmental conditions and material properties on the bistable response of composite laminates. A comprehensive sensitivity analysis of the effects of these factors on curvature behavior was carried out. Uncertainties in the thermal expansion coefficients, Young’s moduli, temperature variation and ply thickness have a significant effect on the bistable behavior of these composites. In another uncertainty analysis, Saberi et al. [32] reported that the transverse thermal expansion coefficient increases the bistability response of the composite laminates. Nevertheless, longitudinal thermal expansion coefficient, moisture content, coefficient of moisture, laminate thickness, and temperature variation reduce the bistability probability. Therefore, rigorous quality-control strategies should be applied during fabrication processes to lessen the discrepancies between the theoretical and experimental.

## 3. Artificial-Intelligence-Based Modeling

Artificial-intelligence-modeling tools may be used as a good alternative to analytical modelling approaches without involving solving complex mathematical equations [33,34,35]. They have high generalization capabilities and have been reported as robust prediction tools [36,37,38]. Moreover, in some cases, analytical approaches fail to obtain accurate solutions due to the multiple assumptions used during the formulation of the problem [39]. For instance, analytical approaches failed to obtain the first three natural frequencies of a BMC and succeeded to obtain only the fourth natural frequency in [40]. Finite element simulations have been also reported as efficient tools to simulate the dynamic behavior of BMCs [10,41]. The integration between artificial-intelligence models and finite-elements tools could help in optimizing the design of bistable structures. 

Saberi et al. [40] developed an ANN model to analysis the vibrational behavior of BMCs. The model was employed to predict the modal parameters of BMCs for stable configurations. The model was trained using datasets extracted from finite element simulations. Natural frequencies were estimated as a function of geometrical parameters using a mathematical formula determined using the developed model. Moreover, physical parameters of the laminate were estimated via optimizing the objective function obtained from the mode-management problem using an advanced metaheuristic optimizer called the firefly algorithm. Chau et al. [42] investigated the design optimization of BMCs using a hybrid model composed of an adaptive neuro-fuzzy inference system (ANFIS), statistical techniques, a fuzzy logic algorithm, a finite element, a desirability function approach, and lightning-attachment-procedure optimization (LAPO). They designed the experiments using statistical techniques and carried out the experiments on a three-dimentianal finite element model. The obtained results, such as displacement, equivalent stress and safety factor, were analyzed using statistical tools. A sensitivity analysis was carried out to refine the design data prior to feeding them into the LAPO algorithm. The desirability values of safety factor and displacement were calculated, and the obtained data were fed into the fuzzy logic algorithm. Then, the ANFIS model was employed to map the design data and a performance characteristic index. Liu et al. [43] developed a machine-learning model to optimize and design a bistable curved beam. The model succeeded in predicting the nonlinear behavior of the beam. The beam structure was optimized based on various objectives, such as forward- and backward-snapping forces as well as stiffness.

Panesar and Weaver [44] employed an ant colony optimizer to enhance the structural behavior of BMCs. Out-of-plane deflection was used as an objective function that should be maximized. The optimizer was used as a subroutine in finite element simulations to obtain the optimal bistable structure as a function of ply angle. Regarding the synthesis of bistable periodic structures, Prasad and Diaz [45] proposed an intelligent topology synthesis approach to two-dimensional bistable periodic structures based on a genetic algorithm and topology optimization. Zhang et al. [46] employed a genetic algorithm technique (NSGA-II) to optimize the inherent compliance of BMC. The main objective of that study was maximizing the advantage of the stiffness variability of the BMC. The proposed model showed high effectiveness and great robustness. It is recommended to apply different metaheuristic methods [47,48] to optimize the performance of artificial-intelligence tools [49,50,51] to predict the behavior of BMC. 

## 4. Actuation Techniques

The actuation of bistable composite structures is an important issue that has been extensively studied in literature. There are many smart materials and mechanisms used to drive that kind of structure, such as piezoelectric actuation, pneumatic actuation, thermal actuation, mechanical actuation, shape memory alloy (SMA) actuation and magnetic actuation.

**Figure 2:**
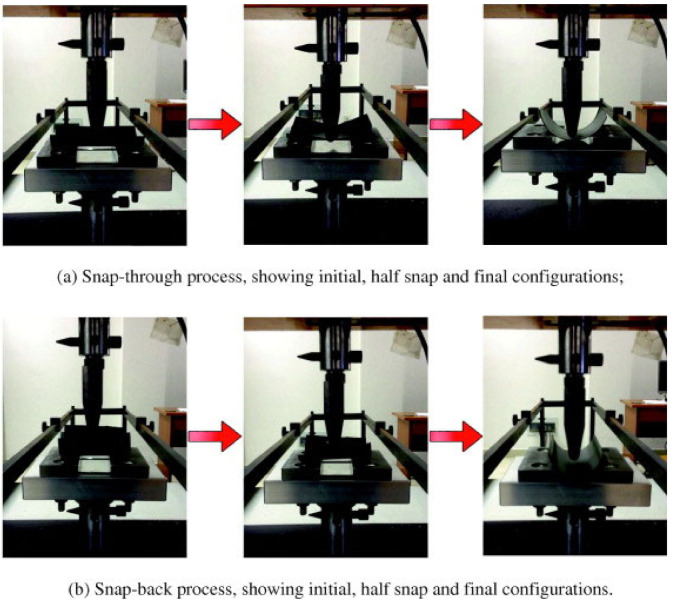
Snap-through and snap-back process using mechanical actuator [52].

Zhang et al. [52] employed a mechanical actuating system to derive bistable composite sheets using a testing machine that provided the sheets with applied forces. The loads were exerted in the middle of the sheet edges to perform the snapping process, as shown in Figure 2. The load–displacement curves were recorded by the testing machine to capture snap loads. The effects of the geometrical and fabrication parameters of the laminate, such as longitudinal length, embrace angle, initial transverse radius, number of plies and ply angle, on the snapping behavior of the laminates were investigated. Among all investigated factors, the ply angle and the mid-plane transverse radius affected the snapping behavior the most. The induced stresses and the snap load increased by increasing the number of plies and the longitudinal length. In another study on mechanical actuating [53], it was reported that the response of a bistable structure could be enhanced via adjusting the elastic modulus ratio between the prestressed strips and mid-layer. Dai et al. [54] employed twisting loading as a mechanical-actuating technique to drive the bistable composite sheets. It was reported that there are three main cure shapes: the paraboloid shape, primary twisted cylindrical shape and snapped cylindrical shape. Moreover, the cured curvature increased by increasing the imperfection. 

Ni et al. [55] developed a multistage pneumatic actuator to control the snapping process of a bistable structure. The actuation process was accomplished using compressed air controlled by a single chip microcomputer. The elapsed snap time required for completing the snap-through process including the controlling time of the pneumatic actuator and the electrical proportion ranging between 0.98 s and 1.25 s. 

Lee et al. [56,57] developed an adaptive piezoelectrically actuated bistable composite that can snap between stable states without any mechanical assistance or continuous energy input. The developed structure was composed of two macro fiber composites bonded together. The released voltage post cure produces in-plane residual stresses that consequently produce the stable states of the structure. That is caused by the applied electric field, which induces an anisotropic strain in the laminates due to the mismatch of their piezoelectric parameters. Kim et al. [58] used a coil-spring actuator made of a shape-memory alloy to control the snapping behavior of a CFRP composite structure. The developed structure succeeded in mimicking the natural behavior of a flytrap leaf with a trapping frequency of 0.046 Hz.

Zhang et al. [59] established an innovative thermally actuated bistable structure composed of bimetallic strips embedded into a hybrid composite laminate. The bimetallic strips have temperature-sensing properties, which enable it to actuate the developed structure. The effects of the geometrical parameters of the laminates and bimetallic strips as well as material properties of the bimetallic strips on the bistable behavior of the hybrid laminates were investigated. Five different bimetallic strips were utilized in that study, namely, aluminum/aluminum, aluminum/zinc, aluminum/steel, aluminum/nickel and aluminum/copper. It was reported that, to increase the produced curvature, the width and thickness of the bimetallic strip as well as the thermal expansion coefficient difference between the strips should be increased. A modified version of this thermally actuated bistable structure utilizing Ni36/Mn75Ni15Cu10 as a bimetallic strip was used in a solar tracking system [60]. 

**Figure 3:**
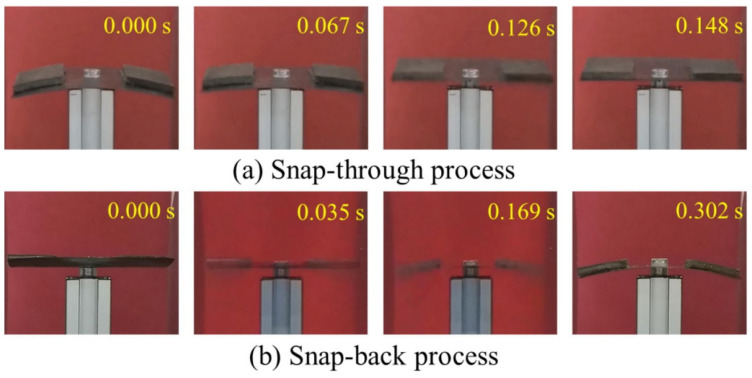
Snap-through and snap-back process using magnetorheological elastomer actuator captured by a digital camera [61].

Zhang et al. [61] developed a novel magnetorheological elastomer to drive an orthogonal bistable composite shell. The elastomer was attached at the edge of a bistable shell using a binder. An external magnetic field was applied to deform the elastomer to produce a magnetic force that was used to actuate the bistable structure. The trigger time required for stable transition was in the order of milliseconds, which reveals the excellent actuation efficiency of the developed elastomer actuator over other actuation techniques. To decrease the response time of the structure the magnetic field should be increased. The snap-back and snap-through process of the bistable structure took 0.302 s and 0.148 s, respectively, as shown in Figure 3.

Recently, Anilkumar et al. [62] developed a BMC controlled by a piezoelectric actuator. The actuators were attached to the middle of the laminate on both sides. Using multiple small actuators instead of a single large one resulted in reducing the out-of-plane displacement. It was reported that distributed piezoelectric actuators can reduce the voltage by up to about 40% during a snap-through process, compared with the concentrated one.

## 5. Energy Harvesting

Harvesting scattered energy and converting it into electrical energy has received great attention in recent years [63,64,65]. There are many energy sources that could be exploited to harvest energy, such as sunlight, mechanical vibration, electromagnetic radiofrequency, thermal gradient, and body motion [66]. Mechanical vibration is induced in various mechanical devices: machinery vibration [67], aeronautical components [68], wind-induced vibration [69], automotive suspension system [70] and flow-induced vibration [71], are the main sources of vibration energy. This kind of energy is suitable for low-power electronic devices due to its profusion and its power density. BMC has shown promising applications in harvesting vibration energy and converting it into electrical energy [15].

Betts et al. [72] investigated the power-generation characteristics and the dynamic response of a bistable asymmetric laminate made of CFRP and equipped with piezoelectric layers attached to a bistable asymmetric laminate. The “snap-through” property of the developed material is exploited to harvest energy, which is consequently used to generate electricity via the attached piezoelectric. The developed system exhibited high levels of extracted power for various levels of applied frequencies. In another study [73], the same authors obtained optimal configurations of bistable composites, such as thickness, aspect ratio, piezoelectric area and stacking sequence, that maximize the generated electrical power. They reported that increasing the size of the piezo-element helps in increasing the receiver area used to harvest energy; but it increases the stiffness of the system which consequently affects the induced stresses and curvature of the laminate. Moreover, increasing the thickness of the laminate has a positive effect on energy generation, as it enables the use of wider piezo-elements.

**Figure 4:**
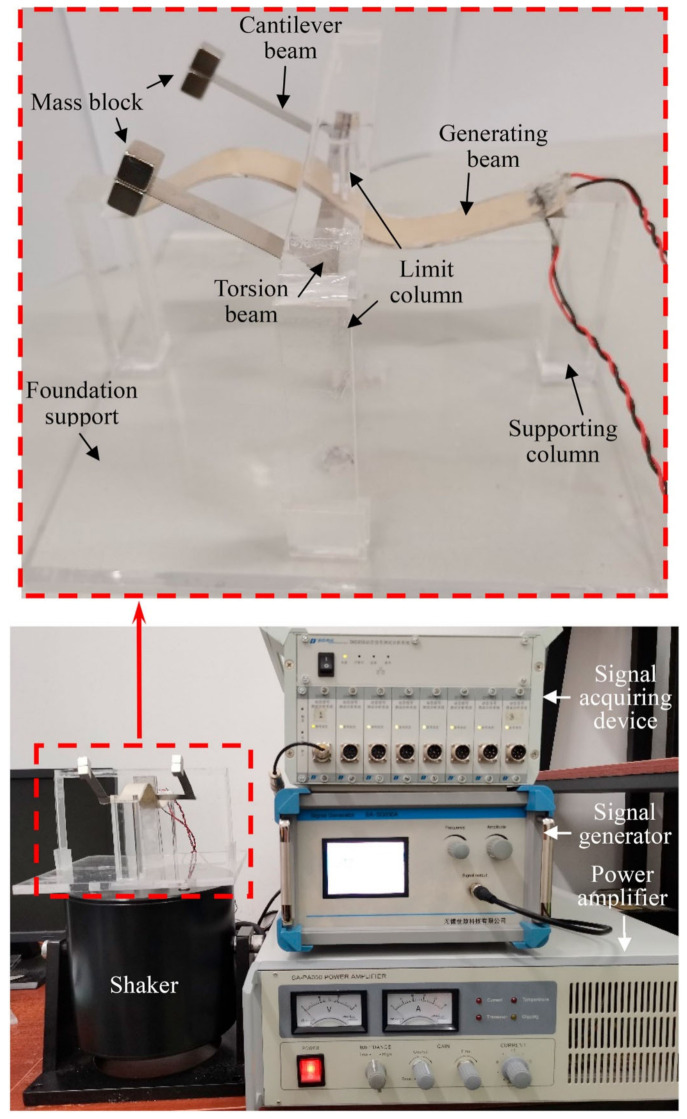
Experimental setup for the validation of the bistable piezoelectric energy harvester [74].

Nan et al. [74] developed a novel bistable piezoelectric system to harvest energy from ambient vibrations, as shown in Figure 4. A pre-deformed beam with a sinusoidal form was used as a host structure on which piezoelectric layers were attached. A torsion device was established on the midspan of the beam to enable effective swinging and to overcome the potential barrier. The harvesting performance of the system was assessed at different operating conditions in terms of the generated power. The optimal output of the harvester was achieved at a load of 47 kΩ, which produced a maximum average-generated power of 0.179 mW.

Lee and Inman [75] experimentally investigated the harvesting performance of the broadband energy of a bistable laminate produced by bonding two macro fiber composites excited using piezoelectric actuation. An electric field was applied on the laminate to produce in-plane residual stresses which resulted in anisotropic strains and switched the laminate from a stable state to another. Different dynamic regimes, such as linear single-well, limit cycle, subharmonic cross-well, chaos, intermittency subharmonic-chaos, intermittency limit cycle–chaos, were tested. The limit cycle regime produced the maximum total power of 130.66 mW at a frequency of 17.5 Hz and optimal load resistance of 39.8 kΩ.

Hybrid vibration-isolation/energy-harvesting systems have received considerable research attention to minimize harmful effects of vibration on the mechanical systems, augment the efficiency of vibration isolation and generate electricity [76,77,78]. Lu et al. [79] used a bistable composite plate with an attached piezoelectric film to harvest energy in a passive isolation of vibration. Analytical modelling of the system based on the virtual-work principle was carried out. They reported that high excitation amplitudes resulted in impairing the performance of the vibration-isolation system at moderate frequencies, decreasing the bandwidth of the vibration isolation and increasing the output voltage at all frequencies. Wu et al. [80] investigated the dynamic behavior of a bistable composite sheet excited using an electromechanical shaker. It was reported that the use of low excitation amplitudes produces a single-well periodic vibration in the composite structure, while the use of high excitations amplitudes produces single-well chaotic vibrations.

**Figure 5:**
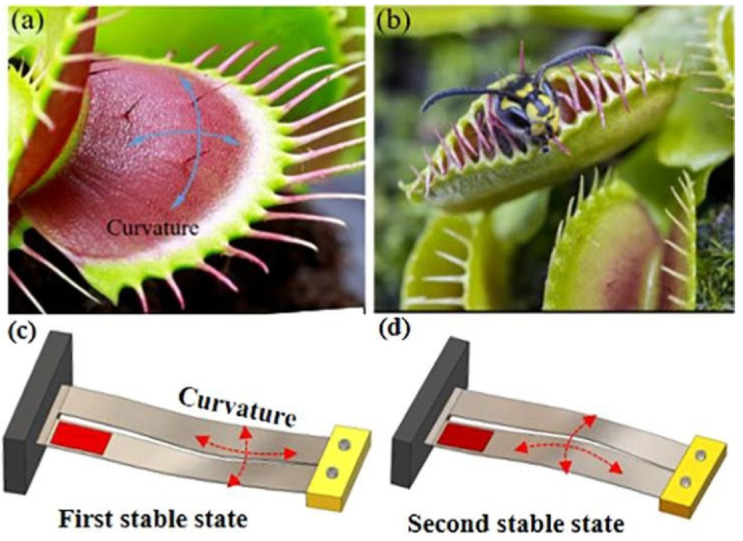
(**a**) First stable state of Venus flytrap leaves; (**b**) second stable state of Venus flytrap leaves; (**c**) first stable state of the designed beams; (**d**) second stable state of the beams [81].

A bio-inspired bistable piezoelectric system was designed to harvest energy from a broadband ambient vibration [81]. That innovative design mimics the rapid shape transition of the double-leaf structure of Venus flytrap leaves, as shown in Figure 5. The snap-through mechanism of this bio-inspired system has two main modes, the first is a local vibration around the stable states under excitations exerted on the fixed base and the second is a global snap between stable states. The designed beams experienced multiple high-frequency vibrations even under harmonic excitation with a single low frequency which enhanced the power generation. The average generated power significantly increased by increasing the excitation level. Qian et al. [82] suggested a novel bio-inspired bi-stable piezoelectric system that mimics fish telemetry tags to harvest energy. The proposed system effectively converted the swing motions of the fish into electrical energy. An average generated power of 1.5 mW was obtained at a frequency of 1.6 Hz and a swing angle of 30°. Wang et al. [67] developed a bio-inspired piezoelectric system for wind-energy harvesting, in which artificial leaves made of polyvinylidene fluoride were developed to mimic the behavior of leaf venation.

In aerospace engineering, bistable structures have been widely used in bistable winglets, variable sweep aerofoils, aircraft landing gear, and variable camber trailing edges. A bistable composite tape-spring has been utilized in hinge-safety assembly used in aircraft landing gear [83], due to its ability to endure large displacements during folding and its viscoelastic constitutive behavior [84]. Wang et al. [85] investigated strain levels induced in the folded state of a bistable composite tape-spring used in an aircraft landing system as a hinge-safety connection. It was reported that three zones were observed in the folded structure, namely, the circular central fold zone; ploy zone; and natural end zone. Extensive changes in transverse curvature and axial strains were observed in the ploy zone. In the fold center, the strain had a uniform distribution across the width. These observations helped the designer to preserve the structural integrity of the bistable composite.

## 6. Conclusions

In this review paper, the applications of bistable morphing composites are discussed. Bistable composites exhibit large deformation between two equilibrium states by applying small energy forces. They can maintain their equilibrium state without any external forces. Different actuating techniques used to control the bistable composites, such as piezoelectric actuation, pneumatic actuation, thermal actuation, mechanical actuation, shape memory alloy actuation and magnetic actuation, are discussed. The mathematical modeling of bistable composites is presented. The bistability response of the composite laminates is highly affected by the thermal expansion coefficient, moisture content, coefficient of moisture, laminate thickness, and temperature variation. Therefore, rigorous quality-control strategies should be applied during the fabrication processes of bistable composites to obtain high-quality structures. Finally, the applications of artificial-intelligence techniques to optimize the design of bitable structures and to predict their response under different actuating schemes are discussed. For future work, it is highly recommended to apply advanced metaheuristic optimizers, such as the jellyfish search algorithm, grey-wolf optimizer and moth-flame optimizer, to optimize the design of bistable structures.

## Data Availability

Not applicable.
